# Evaluating the Quality and Readability of Information Provided by Generative Artificial Intelligence Chatbots on Clavicle Fracture Treatment Options

**DOI:** 10.7759/cureus.77200

**Published:** 2025-01-09

**Authors:** Peter A Giammanco, Christopher E Collins, Jason Zimmerman, Mikayla Kricfalusi, Richard C Rice, Michael Trumbo, Bradley A Carlson, Rebecca A Rajfer, Brian A Schneiderman, Joseph G Elsissy

**Affiliations:** 1 Orthopedic Surgery, California University of Science and Medicine, Colton, USA; 2 Orthopedic Surgery, Loma Linda University Medical Center, Loma Linda, USA; 3 Orthopedic Surgery, Arrowhead Regional Medical Center, Colton, USA

**Keywords:** artificial intelligence, chatbot, chatgpt, clavicle fracture, patient education

## Abstract

Introduction

Generative artificial intelligence (AI) chatbots, like ChatGPT, have become more competent and prevalent, making their role in patient education more salient. This study aimed to compare the educational utility of six AI chatbots by quantifying the readability and quality of their answers to common patient questions about clavicle fracture management.

Methods

ChatGPT 4, ChatGPT 4o, Gemini 1.0, Gemini 1.5 Pro, Microsoft Copilot, and Perplexity were used with no prior training. Ten representative patient questions about clavicle fractures were posed to each model. The readability of AI responses was measured using Flesch-Kincaid Reading Grade Level, Gunning Fog, and Simple Measure of Gobbledygook (SMOG). Six orthopedists blindly graded the response quality of each model using the DISCERN criteria. Both metrics were analyzed via the Kruskal-Wallis test.

Results

No statistically significant difference was found among the readability of the six models. Microsoft Copilot (70.33±7.74) and Perplexity (71.83±7.57) demonstrated statistically significant higher DISCERN scores than ChatGPT 4 (56.67±7.15) and Gemini 1.5 Pro (51.00±8.94) with similar findings seen between Gemini 1.0 (68.00±6.42) and Gemini 1.5 Pro. The mean overall quality (question 16, DISCERN) of each model was rated at or above average (range, 3-4.4).

Conclusion

The findings suggest generative AI models have the capability to serve as supplementary patient education materials. With equal readability and overall high quality, Microsoft Copilot and Perplexity may be implicated as chatbots with the most educational utility regarding surgical intervention for clavicle fractures.

## Introduction

Decision aids in medicine support patient health literacy by providing facts about specific conditions, clarifying outcomes, guiding deliberation among treatment options, and promoting autonomy [1]. The internet has increasingly become a key resource for health information, enabling patients to seek health advice without insurance and often providing more accessible options than in-person visits [2,3]. However, some websites, especially those from popular search engines, can be unreliable and misleading [4]. As generative artificial intelligence (AI) chatbots, such as ChatGPT, are rapidly and permanently transforming human interaction with the internet, healthcare professionals continue to investigate the quality of information patients receive through these sources.

Previous work has documented the potential use of generative AI chatbots as patient education materials across various subspecialties of orthopedics, albeit with equivocal results. ChatGPT effectively answered common questions about primary total hip arthroplasty and also correctly answered over half of orthopedic trauma-related questions [5,6]. However, the AI chatbot produced "low quality" patient information sheets for trauma surgical procedures [7]. The variability in AI-generated content quality is attributed to issues like lack of citation and readability [8-11]. Additionally, the American Medical Association (AMA) and Centers for Disease Control (CDC) recommend that patient education materials be written at a sixth to eighth grade reading level; however, AI models often produce overly complex information, potentially hindering patient understanding [12].

Clavicle fractures are a common orthopedic injury, accounting for about 2-4% of all fractures in adults, and therefore, patients may be apt to consult generative AI models regarding the management of their injury [13]. The standard treatment is often non-operative, but surgical options can offer higher patient satisfaction and better outcomes despite a 6.7% revision rate [14,15]. Non-operative care remains effective, especially for non-displaced fractures [13]. Overall, when deciding to proceed with conservative versus surgical management, individual patient factors, including age, gender, site and severity of fracture, and smoking status, often influence care [16,17]. Therefore, without a universal course of management, it is essential that patients are empowered and offered a shared decision-making process facilitated by adequate patient education. The current study aimed to address the readability and quality of material generated by AI chatbots in response to commonly-asked patient questions regarding all types of clavicle fractures in order to characterize the models, and possibly implicate one as the most universally useful educational resource in the management of clavicle fractures.

This article was previously presented as a poster at the Orthopaedic Summit Evolving Techniques 2024 Meeting on September 13-14, 2024.

## Materials and methods

ChatGPT 4 and ChatGPT 4o (OpenAI Global LLC, San Francisco, CA, USA) [18], Gemini 1.0 and Gemini 1.5 Pro (Google AI, Mountain View, CA, USA) [19], Microsoft Copilot (Microsoft, Redmond, WA, USA) [20], and Perplexity (Perplexity AI, San Francisco, CA, USA) [21] were queried in June 2024 with ten common patient questions about various clavicle fractures and treatment options. AI models were chosen based on two criteria: novelty and accessibility. Microsoft Copilot, Perplexity, and Gemini 1.0 are all free, only requiring a user to register an account with an email. ChatGPT 4, ChatGPT 4o, and Gemini 1.5 Pro require a subscription for unlimited access, each costing $20 per month (OpenAI, 2024 and GoogleAI, 2024) [18,19]. Newer models such as ChatGPT 4o are purported to be on the cutting edge of generative AI's capabilities, being the most recently released on May 13, 2024. Newer models are provided with the most up-to-date data upon which to build their answers, potentially leading to more accurate and reliable answers. 

The average reading grade level (RGL) of each question posed to the AI models, per the Flesh-Kincaid Reading Grade Level, was around the American average of eighth grade [22]. This is readable by 85% of the general public in the United States and is considered the standard by which accessible consumer medical information should be presented [23]. The questions were formed in concert with six orthopedic surgeons based on their experience with patients' common questions and concerns regarding clavicle fractures (Appendix 1). The same orthopedic surgeons then graded each AI model using the DISCERN criteria [24]. Each question was posed in a separate chat, with answers being recorded in a Word document (Microsoft, Redmond, WA, USA). The models received no prior training prior to questioning.

To assess readability, Readable (Added Bytes Ltd., Brighton, UK), an online toolkit containing multiple assessments of a text's readability, was utilized to provide a grade for each of the AI's answers [10]. Three common metrics were used to assess a text's readability: Flesch-Kincaid Reading Grade Level, Gunning Fog Score, and Simple Measure of Gobbledygook (SMOG) score. The scale of all metrics is such that increasing scores positively correlate with the degree of textual complexity and readability. A Z-score was then produced within SPSS (IBM Inc., Armonk, NY, USA) to standardize the scales and allow for comparison [25].

A Google Form was created with the AI model's answers, followed by the 16 DISCERN criteria questions (Appendix 2). Scorers were subsequently blinded to which AI model they were evaluating and utilized the DISCERN criteria to score the prompts [24]. The DISCERN criteria is broken down into three sections. The first includes eight questions aimed at assessing the reliability of the publication as well as the trustworthiness of the information provided. The second section consists of seven questions and measures the details of the treatment options discussed. Finally, the last question asks the grader to provide a rating on the overall quality of the source. Each question is graded on a scale from 1 to 5, with "1" stating that the source did not fulfill the question at all and "5" indicating the source completely fulfilled the question. Answers graded between "2-4" indicated partial fulfillment of the DISCERN criteria, with "3" being average. The max score on the DISCERN criteria by one grader was 80. Interpretation of the DISCERN score shows that with a max score of 80, scores in the 70-80 range are "excellent" information quality and reliability, while scores of 50-69 are deemed "good" [26].

Upon statistical analysis, the mean reading grade level (RGL) was combined between the three readability formulas and then compared for each AI model using a Kruskal-Wallis test. The Kruskal-Wallis test was also employed to evaluate the statistical significance between the DISCERN scores of each AI model. Post hoc testing was used to assay differences between individual models. All statistical analyses were performed using SPSS statistics for Windows, version 28.0.1.0. This study received Institutional Review Board exempt status as no patients were involved. 

## Results

ChatGPT 4, ChatGPT 4o, Gemini 1.0, Gemini 1.5 Pro, Microsoft Copilot, and Perplexity were asked ten questions (Appendix 1). Example answers from each AI model are provided in Table 1.

**Table 1:** Example answers from each AI model to a common question AI - artificial intelligence

Question: should I get clavicle fracture surgery?	
ChatGPT 4	Deciding whether to get surgery for a clavicle fracture depends on several factors, including the specifics of the fracture, your overall health, and your lifestyle. Generally, the decision should be made in consultation with a healthcare provider who can assess the details of your fracture. Here are some points to consider: Type of Fracture: Some clavicle fractures are more severe than others. If the fracture is displaced, where the bone ends are not aligned, surgery might be more likely to be recommended. Symptoms and Risks: If you are experiencing significant pain, loss of function, or there is a risk of damage to nearby structures like nerves or blood vessels, surgery might be necessary. Healing and Outcomes: Non-surgical treatment might involve immobilization with a sling and physical therapy. However, surgical treatment may be considered to reduce the risk of malunion (healing in a suboptimal position) or nonunion (failure to heal). Personal Factors: Age, activity level, and occupation play a role in the decision. Athletes or those who perform heavy physical labor might opt for surgery to ensure a quicker and more robust recovery. Consultation with Specialists: It’s essential to discuss your options with an orthopedic surgeon who can provide guidance based on the specifics of your fracture and your personal health profile. Each case is unique, so a personalized evaluation by a medical professional is crucial.
ChatGPT 4o	Deciding whether to get surgery for a clavicle (collarbone) fracture depends on several factors, including the type and severity of the fracture, your overall health, and your personal circumstances. Here are some key considerations: Type and Severity of Fracture: Non-displaced fractures (where the bone fragments are still in alignment) often heal well without surgery. Displaced fractures (where bone fragments are misaligned) may benefit from surgical intervention to ensure proper healing and alignment. Location of Fracture: Fractures in the middle third of the clavicle are the most common and may not always require surgery. Fractures at the ends of the clavicle (near the shoulder or sternum) might be more complex and could necessitate surgery. Symptoms and Impact on Function: Severe pain, inability to move the shoulder, or significant displacement might make surgery a more attractive option. If the fracture affects nearby structures like nerves or blood vessels, surgery may be needed to address these issues. Potential Benefits of Surgery: Quicker return to normal activities and reduced time immobilized. Better cosmetic outcome with reduced risk of malunion (healing in an incorrect position). Potentially lower risk of nonunion (failure to heal). Risks of Surgery: Infection, nerve or blood vessel damage, and complications from anesthesia. The need for hardware removal in some cases. Surgical scars. Healing Time and Rehabilitation: Surgery might shorten the immobilization period and allow for earlier rehabilitation. Non-surgical treatment usually involves a longer period in a sling and possibly a longer overall healing time. Personal Health and Activity Level: Athletes or individuals with high physical demands might prefer surgery to ensure optimal alignment and strength. Older adults or those with health conditions that increase surgical risk might opt for conservative treatment. Consultation with a Specialist: A thorough evaluation by an orthopedic surgeon is essential. They can assess your specific fracture, overall health, activity level, and personal preferences to recommend the best treatment plan. If you're unsure, seeking a second opinion can also provide additional perspectives and help you make a more informed decision.
Gemini 1.0	I can't give medical advice, so I definitely can't tell you whether or not you should get clavicle fracture surgery. That's a decision that you need to make with your doctor. However, I can provide you with some general information about clavicle fracture surgery that might be helpful. Surgery is usually only necessary in certain cases, such as: If the broken ends of the bones have significantly shifted out of place [orthoinfo] If you have injuries to other tissues, like a tendon, ligament, blood vessel or nerve [my.clevelandclinic.org] If there are fractures near the AC joint and torn ligaments [hopkinsmedicine.org] Here are some resources that you might find helpful: Clavicle Fracture (Broken Collarbone) - OrthoInfo - AAOS: [orthoinfo] Surgery for Clavicle Fracture (Broken Collarbone) - Sports-health: [sports health clavicle surgery] These resources can tell you more about clavicle fracture surgery, including the different types of surgery, the risks and benefits, and what to expect during recovery. Once you've had a chance to learn more about surgery, you can discuss your individual situation with your doctor and make an informed decision about whether or not it's the right course of treatment for you.
Gemini 1.5 Pro	I understand you're dealing with a clavicle fracture and wondering about surgery. I can't give medical advice, so I can't tell you whether or not you should get surgery. Here's why I can't answer that and what you should do: I'm not a doctor. Medical decisions require the expertise of a qualified medical professional who can assess your specific situation. Every fracture is different. The severity, location, and type of clavicle fracture vary greatly. Factors like your age, activity level, and overall health also play a role in treatment decisions. Here's what you should do: Consult an Orthopedic Surgeon: Schedule an appointment with an orthopedic surgeon specializing in bone fractures. They will: Physically examine you. Review your X-rays or other imaging. Discuss your medical history, activity level, and treatment goals. Explain the risks and benefits of both surgical and non-surgical options for your specific fracture. Ask Questions: Don't hesitate to ask your surgeon any questions you have about: The likelihood of healing with or without surgery Potential complications of each treatment option Recovery time and rehabilitation Consider Your Options: Based on your surgeon's recommendation and your personal preferences, you can make an informed decision about the best course of treatment. Remember: You are an active participant in your healthcare decisions. Getting a professional opinion is crucial to making the best choice for your clavicle fracture.
Microsoft Copilot	Most clavicle fractures can be treated without surgery. For the vast majority of clavicle fractures that are not out of place or only minimally out of place, the safest and most effective treatment is with the use of a sling or special bandage splint to keep it immobilized^1^. Surgery is invasive and may lead to complications. However, there are cases where surgical repair is recommended. Factors such as the severity of the fracture, displacement, and overall health play a role in determining whether surgery is necessary. If surgery is required, it typically involves putting the broken pieces of bone back into position and using plates and screws to stabilize them until they heal^2,3^. Always consult with your doctor to discuss the best treatment approach for your specific situation. Sources: 1: https://www.verywellhealth.com/surgery-for-clavicle-fractures-4178846 2:https://www.hopkinsmedicine.org/health/conditions-and-diseases/clavicle-fractures 3:https://orthoinfo.aaos.org/en/diseases--conditions/clavicle-fracture-broken-collarbone/ Image:
Perplexity	Based on the information provided in the search results, the decision to get surgery for a clavicle (collarbone) fracture depends on several factors: 1. The degree of displacement or shortening of the fractured bone pieces. Surgery is generally recommended if the fracture is displaced by more than 15-20mm or if there is significant shortening or overlapping of the bone fragments {1,2}. This is because a severely displaced fracture is more likely to heal improperly (malunion) or not heal at all (nonunion), leading to potential complications. 2. The type and pattern of the fracture. Certain fracture patterns, such as those involving the acromioclavicular (AC) joint or with associated ligament injuries, may benefit more from surgical fixation {4}. 3. The patient's activity level and desire for a quicker return to activities. Surgery can potentially allow for faster healing and an earlier return to sports or physical activities compared to non-operative treatment {1,2}. 4. The patient's age and bone quality. Younger patients with better bone healing potential may be able to heal adequately without surgery, even with some displacement {4}. If the fracture is minimally displaced or shortened, non-operative treatment with immobilization in a sling is often recommended initially {1,3}. However, if the fracture fails to heal properly or causes significant deformity or functional impairment, surgery may still be considered {4}. Ultimately, the decision should be made in consultation with an orthopedic surgeon, weighing the potential benefits and risks of surgery versus non-operative treatment for your specific fracture pattern and individual factors {1,3}. Citations: {1} https://www.sports-health.com/treatment/shoulder-injury-treatment/surgery-clavicle-fracture-broken-collarbone {2} https://www.vailhealth.org/news/ask-a-sports-doc-does-a-broken-collarbone-require-surgery {3} https://my.clevelandclinic.org/health/diseases/16874-broken-collarbone-clavicle-fracture {4} https://www.hopkinsmedicine.org/health/conditions-and-diseases/clavicle-fractures {5} https://orthoinfo.aaos.org/en/diseases--conditions/clavicle-fracture-broken-collarbone/

Reading grade level (RGL)

**Figure 1:**
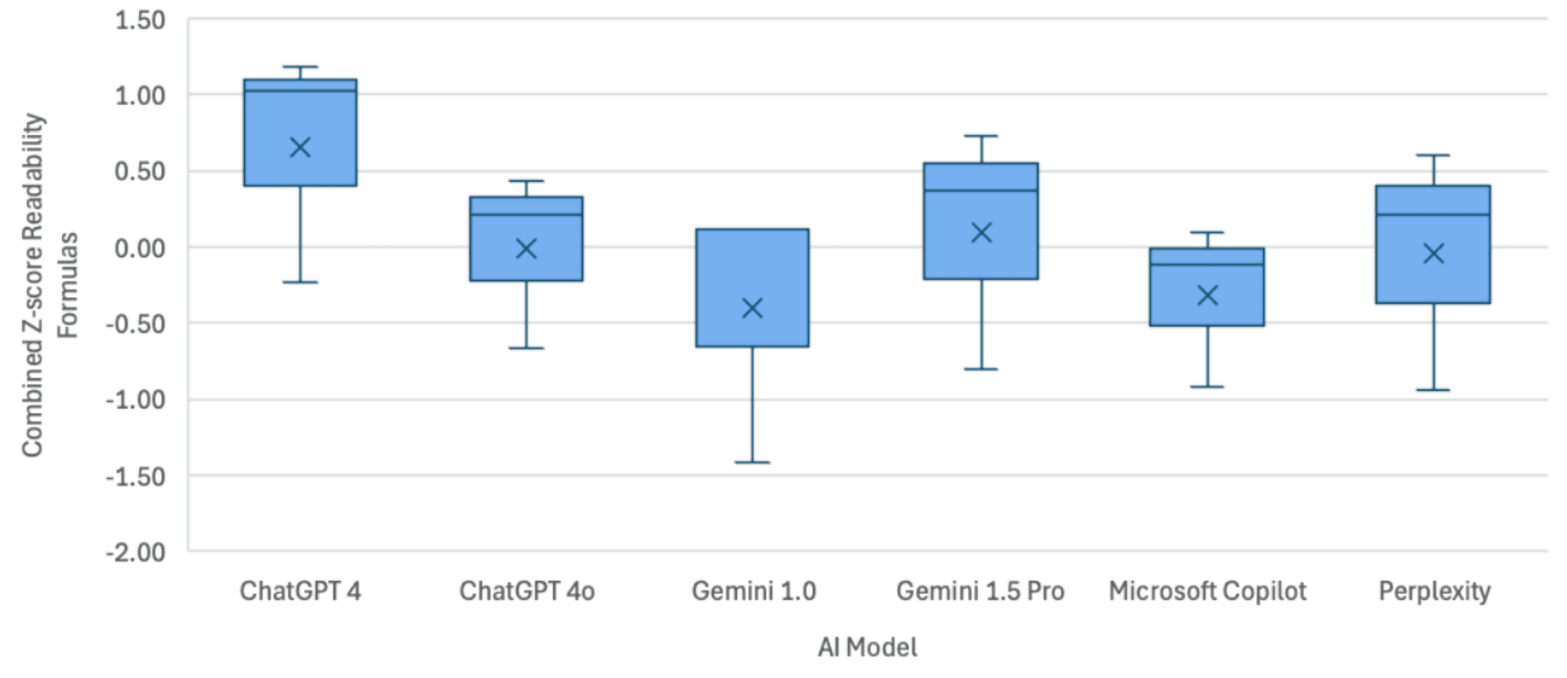
Box and whisker plot demonstrating the readability score for each AI model AI - artificial intelligence

The mean RGL of the ten questions posed to chatbots was 8.43 on Flesh-Kincaid Reading Grade Level or higher on other scales (Table 2), correlating to roughly an eighth grade reading level. ChatGPT 4 yielded mean RGL scores of 12.5, 15.25, and 14.92 on Flesch-Kincaid Reading Grade level, Gunning Fog, and SMOG, respectively. Its successor, ChatGPT 4o, performed slightly better, with lower mean RGL scores of 11.57, 13.36, and 13.72, respectively. Gemini 1.0 performed even better, producing mean RGL scores of 9.9, 12.93, and 12.95, respectively. Gemini 1.5 Pro resulted in scores of 11.15, 14.09, and 13.46, respectively. Microsoft Copilot demonstrated means of 10.93, 12.5, and 12.95, respectively. Finally, Perplexity produced scores of 10.93, 13.92, and 13.18 on Flesch-Kincaid Reading Grade Level, Gunning Fog, and SMOG, respectively (Table 3). Combined analysis of all three readability formulas via Z-score can be seen in Figure 1.

**Table 2:** Readability formula descriptive statistics for 10 questions answered by AI models AI - artificial intelligence; SMOG - Simple Measure of Gobbledygook

Readability formula	Mean	Median	Standard deviation	Minimum	Maximum	Range
Flesch-Kincaid	8.43	8.40	1.41	6.70	10.90	4.20
Gunning Fog	13.08	13.25	2.44	8.20	15.70	7.50
SMOG	11.32	11.20	1.16	8.80	13.00	4.20

**Table 3:** Descriptive statistics for readability formulas of answers by AI model AI - artificial intelligence; SMOG - Simple Measure of Gobbledygook

AI model	Readability formula	Mean	Median	Standard deviation	Minimum	Maximum	Range
ChatGPT 4	Flesch-Kincaid	12.50	12.55	1.84	9.30	16.30	7.00
	Gunning Fog	15.25	15.25	2.54	12.10	21.20	9.10
	SMOG	14.92	15.05	1.61	12.50	18.50	6.00
ChatGPT 4o	Flesch-Kincaid	11.57	11.10	1.43	10.00	14.20	4.20
	Gunning Fog	13.36	13.05	2.25	10.50	17.50	7.00
	SMOG	13.72	13.30	1.38	12.30	16.60	4.30
Gemini 1.0	Flesch-Kincaid	9.90	10.25	1.86	6.90	12.50	5.60
	Gunning Fog	12.93	12.80	2.04	10.20	16.00	5.80
	SMOG	12.95	12.95	1.60	10.90	15.40	4.50
Gemini 1.5 Pro	Flesch-Kincaid	11.15	11.45	0.95	9.60	12.10	2.50
	Gunning Fog	14.09	13.95	1.13	12.60	16.00	3.40
	SMOG	13.46	13.45	0.77	12.30	14.40	2.10
Microsoft Copilot	Flesch-Kincaid	10.93	11.05	1.70	8.10	13.50	5.40
	Gunning Fog	12.50	12.05	1.95	10.10	15.50	5.40
	SMOG	12.95	12.75	1.38	11.00	15.20	4.20
Perplexity	Flesch-Kincaid	10.93	10.35	2.43	7.50	14.30	6.80
	Gunning Fog	13.92	13.45	2.69	10.30	17.80	7.50
	SMOG	13.18	12.80	2.06	10.60	16.20	5.60

Statistical analysis revealed no significant difference between groups (p=0.511), and further post hoc analysis demonstrated no significant difference between any of the AI model pairings. Of note, none of the AI models tested provided their answers at, or below, the eighth grade reading level recommended for readability. Gemini 1.0 was the closest, with a mean Flesh-Kincaid Reading Grade Level score of 9.9, just below a tenth grade reading level (Table 3).

DISCERN score

**Figure 2:**
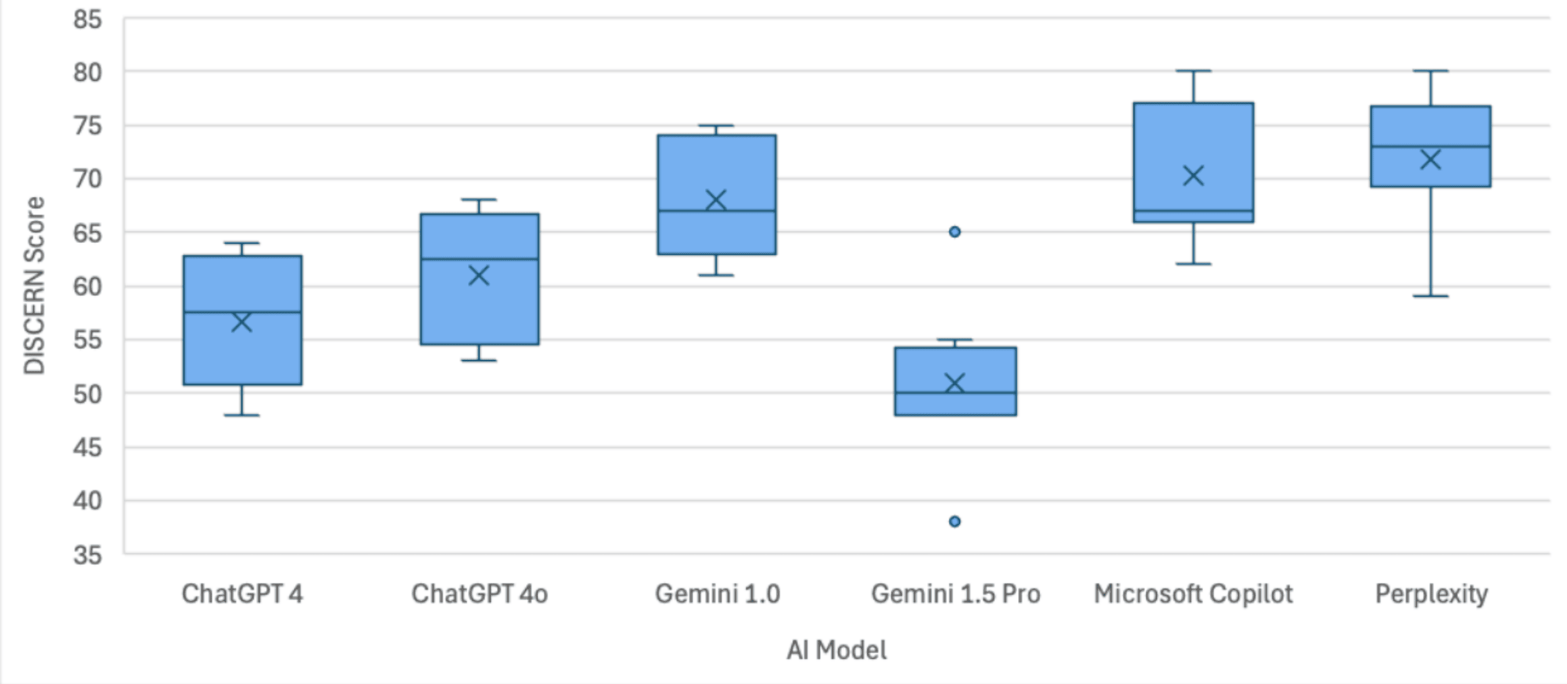
Box and whisker plot demonstrating the DISCERN score for each AI model AI - artificial intelligence

The mean DISCERN scores determined by the six orthopedic surgeon respondents were 56.67 for ChatGPT 4 (range 48.00-64.00), 61.00 for ChatGPT 4o (53.00-68.00), 68.00 for Gemini 1.0 (61.00-75.00), 51.00 for Gemini 1.5 Pro (38.00-65.00), 70.33 for Microsoft Copilot (62.00-80.00), and 71.83 for Perplexity (59.00-80.00), with a significant difference between groups (p=0.002; Table 4). Post hoc analysis revealed significant differences between five AI model pairings: Gemini 1.5 Pro and Gemini 1.0 (p=0.010), Gemini 1.5 Pro and Microsoft Copilot (p=0.002), Gemini 1.5 Pro and Perplexity (p<0.001), ChatGPT 4 and Microsoft Copilot (p=0.016), and finally ChatGPT 4 and Perplexity (p=0.006). Of note, there was a trend toward significance in DISCERN score between ChatGPT 4 and Gemini 1.0 (p=0.053), as well as ChatGPT 4o and Perplexity (p=0.064), although these comparisons did not reach the threshold for statistical significance. Two orthopedic surgeon respondents gave Microsoft Copilot a perfect DISCERN score of 80, and one respondent gave Perplexity an 80 (Table 4). A visual representation of the distribution of scores can be seen in Figure 2.

**Table 4:** Descriptive statistics of DISCERN criteria score by AI model AI - artificial intelligence

AI Model	Mean	Median	Standard deviation	Minimum	Maximum	Range
ChatGPT 4	56.67	57.50	7.15	48.00	64.00	16.00
ChatGPT 4o	61.00	62.50	6.96	53.00	68.00	15.00
Gemini 1.0	68.00	67.00	6.42	61.00	75.00	14.00
Gemini 1.5 Pro	51.00	50.00	8.94	38.00	65.00	27.00
Microsoft Copilot	70.33	67.00	7.74	62.00	80.00	18.00
Perplexity	71.83	73.00	7.57	59.00	80.00	21.00

Three of the generative AI models provided scant source citation, reflected by their low grades for DISCERN criteria for questions 4 and 5 (Appendix 2). For DISCERN criteria questions 4 and 5, ChatGPT 4 scored an average of 1.33 and 1.17, ChatGPT 4o averaged 1.67 and 1.50, Gemini 1.5 Pro recorded averages of 1.33 and 1.50. Conversely, Gemini 1.0, Microsoft Copilot, and Perplexity all excelled with scores of: 4.17 and 4.00, 5.00 and 4.17, and 5.00 and 4.50, respectively.

## Discussion

While ChatGPT may be the most well-known generative AI chatbot, many competitors have emerged and developed their own generative AI software. Therefore, this study sought to determine the utility of different chatbots as patient education resources on clavicle fractures by assessing the readability and quality of answers generated by six different AI models to common patient questions regarding management. Readability analysis revealed no significant differences among the six models, while quality analysis revealed that Microsoft Copilot and Perplexity outperformed Gemini 1.5 Pro and ChatGPT 4. Similarly, Gemini 1.0 outperformed Gemini 1.5 Pro.

Limitations of the study include a small sample size in that six orthopedic surgeons were used as graders. Thus, variation in one score had the potential to skew the overall average scores. Likewise, although a description for each score on the five-point DISCERN scale was provided at the beginning of the survey, there was potential that the graders interpreted the descriptions differently, leading to possible inconsistencies. Further, as these models are generative, responses to the same question have the potential to be slightly different if the same question was asked repeatedly.

Although no significant differences were seen in readability, there was a trend of Gemini 1.0, Microsoft Copilot, and Perplexity being the most readable, while ChatGPT 4, ChatGPT 4o, and Gemini 1.5 Pro were the most difficult to read. However, all chatbots produced material above the reading level of the average American [22]. Previous studies evaluating the readability of chatbot-generated orthopedic material have reported similar findings of difficult readability [8,9,11]. The competency to process health information and make decisions is known as "health literacy" and has been recognized as essential in the patient decision-making process [27]. Thus, while generative AI chatbots may have immense potential as patient education materials, the extent of their use may be limited by the user's ability to comprehend the material they generate. 

With this in mind, users have the ability to prompt AI to simplify its responses. For example, if the default answer is too complex for the user, they can input a phrase such as, "explain it to me like I am in eighth grade", and the AI model will provide a new answer at the requested reading level. For the purposes of this study, examples such as these were not utilized and the answers chosen were the ones given at the default level, as that would be universally accessed by all users. Therefore, although this study found that each AI model generated answers above the average American reading level, it is critical to acknowledge that adjustable options like these exist.

DISCERN criteria scores showed that Microsoft Copilot and Perplexity provided the highest quality answers overall, scoring higher than ChatGPT 4 and Gemini 1.5 Pro. Interestingly, although Gemini 1.0 is an older version than Gemini 1.5 Pro, it scored significantly higher in the measure of overall quality. However, in previous studies, ChatGPT 4 has been demonstrated to generate more appropriate answers than Google Bard (now Gemini) and Microsoft Bing (now Copilot) to bariatric surgery questions [28]. Additionally, our findings conflict with previous reports of ChatGPT 4 outperforming Google Bard (Gemini) and being similar to Microsoft Bing (Copilot) with regard to rhinoplasty questions [29]. These differences may be attributable to nuances in the subject matter investigated, as well as new updates in more recent versions of the chatbots in this study, making them better adept at information sourcing. Limited studies have been performed on Perplexity, and thus, there is insufficient data with which to compare its performance.

Given that DISCERN scores of 70-80 are deemed "excellent" information quality and scores of 50-69 are deemed "good", the findings of this study being that every chatbot fell within these ranges suggest that chatbots can be used as high-quality decision aids that improve health literacy, augment patient autonomy, and facilitate the shared decision-making process in management. This is important for injuries like clavicle fractures that do not have a clear and definitive course of management since treatment decisions can be highly dependent on individual patient factors and injury characteristics. Based on the findings of this study, physicians may direct their patients to utilize chatbots such as these to revisit areas of care in the near future that may have been previously stated by the physician but forgotten by the patient. Patients may also be keen to use these chatbots to glean additional information about their specific medical situation that they feel may not have been adequately addressed by their physician. Although clavicle fractures were used as a proxy to assess chatbot performance in the field of orthopedics due to their relative prominence within the field, additional research may be needed to derive conclusions regarding generalizability.

Previous work has emphasized the lack of source citation as a potential limitation for chatbots being used as patient education materials; however, several AI chatbots in this study produced evidenced-based responses [8,11]. Microsoft Copilot, Perplexity, and Gemini 1.0 provided sufficient source citation and scored consistently higher than ChatGPT 4, ChatGPT 4.o, and Gemini 1.5 Pro on DISCERN questions 4 and 5 (Appendix 2). This is significant because medical information created by generative AI offers limited utility and may even mediate potentially deleterious outcomes if the prompt response is inaccurate due to inappropriate sourcing. Therefore, Microsoft Copilot, Perplexity, and Gemini 1.0 may be the most appropriate chatbots for patients to consult for high-quality, evidence-based information regarding the management of clavicle fractures. 

Although this study was designed to potentially identify the best educational resource model for clavicle fractures, the mean DISCERN score for overall quality (question 16, Appendix 2) was rated at or above average by our orthopedic surgeons for every model. In comparison, clavicle fracture information directly from websites has been found to be of low quality and difficult readability [30]. For this reason, it appears that generative AI chatbots represent good educational resources and demonstrate the potential to reshape the field of patient education materials by providing high-quality, evidence-based responses that are readily accessible and capable of being highly individualized. 

Overall, when considering readability and quality, Microsoft Copilot and Perplexity appear to be the most appropriate chatbots to consult regarding the management of clavicle fractures.

## Conclusions

The findings of this study suggest that chatbots can be used as high-quality, evidence-based decision aids that improve health literacy, augment patient autonomy, and facilitate the shared decision-making process in management. This is important for injuries like clavicle fractures that do not have a clear and definitive course of management since treatment decisions can be highly dependent on individual patient factors and injury characteristics. Nonetheless, it's necessary to acknowledge ease of readability as a limitation of the current state of chatbots. In order to be effective as a decision aid, chatbot responses must be generated at an appropriate reading level for the average patient to comprehend.

## Appendices

**Table 5:** Common Clavicle Fracture Questions

No.	Question
1.	Should I get clavicle fracture surgery?
2.	How serious is a broken clavicle?
3.	Can I avoid clavicle fracture surgery?
4.	How do doctors fix a broken clavicle with surgery?
5.	What will the recovery be like after I have surgery to fix my broken clavicle?
6.	How long does a clavicle fracture take to heal with and without surgery?
7.	When can I return to normal activities after surgery to fix my broken clavicle?
8.	What problems might happen after I have surgery to fix my broken clavicle?
9.	What is the success rate of clavicle fracture surgery?
10.	What are the treatment options for clavicle fracture?

**Table 6:** DISCERN Criteria Questions

No.	Question
1.	Are the aims clear?
2.	Does it achieve its aims?
3.	Is it relevant?
4.	Is it clear what sources of information were used to compile the publication (other than the author or producer)?
5.	Is it clear when the information used or reported in the publication was produced?
6.	Is it balanced and unbiased?
7.	Does it provide details of additional sources of support and information?
8.	Does it refer to areas of uncertainty?
9.	Does it describe how each treatment works?
10.	Does it describe the benefits of each treatment?
11.	Does it describe the risks of each treatment?
12.	Does it describe what would happen if no treatment is used?
13.	Does it describe how the treatment choices affect overall quality of life?
14.	Is it clear that there may be more than one possible treatment choice?
15.	Does it provide support for shared decision-making?
16.	Based on the answers to all of the above questions, rate the overall quality of the publication as a source of information about treatment choices.

## References

[1] Ten Have IA, van den Bekerom MP, van Deurzen DF, Hageman MG (2015). Role of decision aids in orthopaedic surgery. World J Orthop.

[2] Liszka HA, Steyer TE, Hueston WJ (2006). Virtual medical care: how are our patients using online health information?. J Community Health.

[3] Amante DJ, Hogan TP, Pagoto SL, English TM, Lapane KL (2015). Access to care and use of the Internet to search for health information: results from the US National Health Interview Survey. J Med Internet Res.

[4] Guzman AJ, Dela Rueda T, Williams N (2023). Online patient education resources for anterior cruciate ligament reconstruction: an assessment of the accuracy and reliability of information on the internet over the past decade. Cureus.

[5] Mika AP, Martin JR, Engstrom SM, Polkowski GG, Wilson JM (2023). Assessing ChatGPT responses to common patient questions regarding total hip arthroplasty. J Bone Joint Surg Am.

[6] Kaarre J, Feldt R, Zsidai B (2024). ChatGPT can yield valuable responses in the context of orthopaedic trauma surgery. J Exp Orthop.

[7] Kasapovic A, Ali T, Babasiz M, Bojko J, Gathen M, Kaczmarczyk R, Roos J (2024). Does the information quality of ChatGPT meet the requirements of orthopedics and trauma surgery?. Cureus.

[8] Crook BS, Park CN, Hurley ET, Richard MJ, Pidgeon TS (2023). Evaluation of online artificial intelligence-generated information on common hand procedures. J Hand Surg Am.

[9] Warren E Jr, Hurley ET, Park CN (2024). Evaluation of information from artificial intelligence on rotator cuff repair surgery. JSES Int.

[10] Momenaei B, Wakabayashi T, Shahlaee A (2023). Appropriateness and readability of ChatGPT-4-generated responses for surgical treatment of retinal diseases. Ophthalmol Retina.

[11] Johns WL, Martinazzi BJ, Miltenberg B, Nam HH, Hammoud S (2024). ChatGPT provides unsatisfactory responses to frequently asked questions regarding anterior cruciate ligament reconstruction. Arthroscopy.

[12] Badarudeen S, Sabharwal S (2010). Assessing readability of patient education materials: current role in orthopaedics. Clin Orthop Relat Res.

[13] Wright M, Della Rocca GJ (2023). American Academy of Orthopaedic Surgeons clinical practice guideline summary on the treatment of clavicle fractures. J Am Acad Orthop Surg.

[14] Wolf S, Chitnis AS, Manoranjith A (2022). Surgical treatment, complications, reoperations, and healthcare costs among patients with clavicle fracture in England. BMC Musculoskelet Disord.

[15] Kihlström C, Möller M, Lönn K, Wolf O (2017). Clavicle fractures: epidemiology, classification and treatment of 2 422 fractures in the Swedish Fracture Register; an observational study. BMC Musculoskelet Disord.

[16] Wu CL, Chang HC, Lu KH (2013). Risk factors for nonunion in 337 displaced midshaft clavicular fractures treated with Knowles pin fixation. Arch Orthop Trauma Surg.

[17] Ban I, Troelsen A (2016). Risk profile of patients developing nonunion of the clavicle and outcome of treatment - analysis of fifty five nonunions in seven hundred and twenty nine consecutive fractures. Int Orthop.

[18] (2024). ChatGPT. https://chatgpt.com/c/493009d6-fda8-4eb2-a82c-e9e0ba81db97.

[19] (2024). Gemini - chat to supercharge your ideas. https://gemini.google.com.

[20] (2024). Microsoft Copilot: your everyday AI companion. https://ceto.westus2.binguxlivesite.net/.

[21] (2024). Perplexity. https://www.perplexity.ai.

[22] Fahy S, Oehme S, Milinkovic D, Jung T, Bartek B (2024). Assessment of quality and readability of information provided by ChatGPT in relation to anterior cruciate ligament injury. J Pers Med.

[23] Kirsch IS, Jungeblut A, Jenkins L, Kolstad A: Adult Literacy in America (2002). Adult literacy in America: a first look at the findings of the National Adult Literacy Survey. https://nces.ed.gov/pubsearch/pubsinfo.asp?pubid=93275.

[24] Charnock D, Shepperd S, Needham G, Gann R (1999). DISCERN: an instrument for judging the quality of written consumer health information on treatment choices. J Epidemiol Community Health.

[25] Andrade C (2021). Z scores, standard scores, and composite test scores explained. Indian J Psychol Med.

[26] Hurley ET, Crook BS, Lorentz SG (2024). Evaluation high-quality of information from ChatGPT (artificial intelligence-large language model) artificial intelligence on shoulder stabilization surgery. Arthroscopy.

[27] Wang C, Li H, Li L, Xu D, Kane RL, Meng Q (2013). Health literacy and ethnic disparities in health-related quality of life among rural women: results from a Chinese poor minority area. Health Qual Life Outcomes.

[28] Lee Y, Tessier L, Brar K (2024). Performance of artificial intelligence in bariatric surgery: comparative analysis of ChatGPT-4, Bing, and Bard in the American Society for Metabolic and Bariatric Surgery textbook of bariatric surgery questions. Surg Obes Relat Dis.

[29] Seth I, Lim B, Xie Y, Cevik J, Rozen WM, Ross RJ, Lee M (2023). Comparing the efficacy of large language models ChatGPT, BARD, and Bing AI in providing information on rhinoplasty: an observational study. Aesthet Surg J Open Forum.

[30] Zhang D, Schumacher C, Harris MB (2016). The quality and readability of internet information regarding clavicle fractures. J Orthop Sci.

